# A Medical Consultation Service on Facebook: Descriptive Analysis of Questions Answered

**DOI:** 10.2196/jmir.3194

**Published:** 2014-09-04

**Authors:** Otto Helve

**Affiliations:** ^1^Children's HospitalHelsinki University Central HospitalUniversity of HelsinkiHelsinkiFinland

**Keywords:** social media, Internet, health information, health promotion

## Abstract

**Background:**

Social media is used increasingly by the general public to access health information. However, a lack of models for health information distribution limits the presence of publicly funded services on social media sites.

**Objective:**

The goal of the study was to present a model for delivering child health information to parents through a social media site.

**Methods:**

A Facebook site was launched for 11 months based on a question-and-answer service produced by a pediatrician and open to Facebook users over 18 years old. If the answer did not include a further referral to a health care service provider, the question was considered comprehensively answered. The site was funded by a pharmaceutical company, and it included an advertisement of a pharmaceutical product for children’s fever and pain.

**Results:**

During the study, 768 questions were submitted: an average of 69.8 (SD 31.7) per month. There were 245,533 independent Facebook users on the site, with an average of 727.0 (SD 2280.6) per day. Infections were the most common theme in questions (355/768, 46.2%). Questions were more likely to be comprehensively answered if they were related to infections (279/355, 78.6%) than questions related to non-infectious symptoms (265/423, 64.2%, *P*=.003).

**Conclusions:**

On this site aimed at parents of small children, personalized answers were an effective way of delivering information. The service is likely to have reduced the need for further contacts with a health care service provider in more than half of the cases. The site could serve as a model for publicly funded health information distribution.

## Introduction

Social media has changed the way the general public accesses health information [1]. With the increasing use of handheld mobile devices, health information may be readily available to an even larger public, as seen in the United States [2]. Facebook, the social media tool with the widest active user base, has 1.28 billion users as of March 31, 2014 [3]. Health information is distributed and discussed on many social media tools, and one in five Americans uses social media as a source of health care information [4]. Of Facebook users in the United States, 94% have used Facebook to gather information on their health [4].

However, user-generated health content on social media is generally inconsistent with clinical guidelines and professional knowledge, and non-biased information can be difficult to obtain [5]. Publicly funded services and institutes have a limited role in distributing health information on social media [6], possibly due to a lack of models for using social media in health information promotion [7,8]. In Finland, information on child health is traditionally delivered by “well-baby” clinics, which offer both general information on health care and address individual needs. At the moment, most of the information is delivered during patient visits and by telephone consultation. The clinics are understaffed with regard to the national recommendations, and this is reflected in their capacity to deliver health information [9]. It is evident that methods to reach a larger population are needed to meet the demand. As in the United States, although no such data exist, it is likely that the use of social media for seeking health information is increasing also in Finland. Stroever et al [10] found that social media was an effective way to communicate child health information to low-income parents. Furthermore, the information was considered reliable if distributed by perceived experts.

While social media can be used to distribute specific information on issues addressed by individual Internet users, these discussions can be accessed by countless others on an open site, making the information available to a larger audience. Depicted in our study is a social media site for distributing child health information to parents. The information was distributed by a pediatrician. The aim was to determine the number of questions generated by the site, the profile of parents’ concerns, and the number of visitors on the site. Also, the parental contact was assessed by evaluating whether or not the answers to questions included further referral to a health care provider.

## Methods

### Material

**Figure 1:**
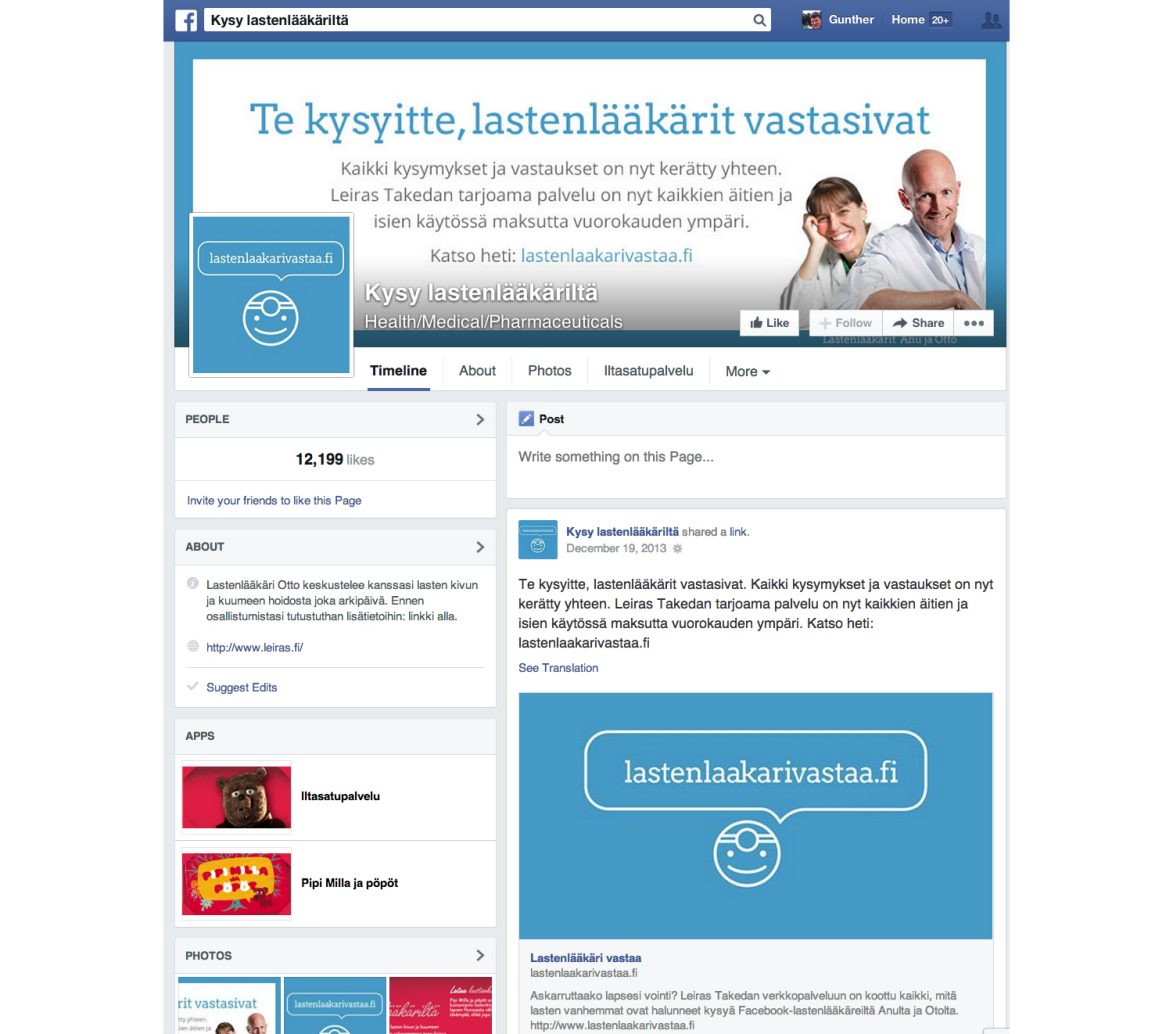
Screenshot of Kysylastenlaakarilta Facebook page.

A Facebook site, Kysy lastenlääkäriltä (“Ask a pediatrician”; Figure 1) based on a question-and-answer service produced by a pediatrician (the author) was established in September 2010. The site was funded by a Finnish pharmaceutical company and included an advertisement for acetaminophen (paracetamol), a pharmaceutical product for children’s fever and pain. The company had no control over issues discussed on the site or answers given by the pediatrician. The site was run in Finnish and advertised in national advertisement campaigns in magazines aimed at parents of small children. The service was open to all Facebook users aged over 18 years.

The local ethics committee approved the study.

### Methodology

A pilot service was initiated at 2- to 3-week irregular intervals depending on demand between September 2010 and March 2011. This experience showed that the demand for a more continuous service was substantial. Therefore, the service was launched for a continuous 7-month period from September 2011, including a 2-week Christmas closure. During the closure, the Facebook site remained open so that previously written questions and answers could be seen by visitors, but no new questions were answered. After a summer closure from April to the end of August 2012, the service was opened again in September until December 14, 2012 (4 months). The results from these 11 months are used in this analysis.

The service was intended to answer questions on children’s fever and pain, but parental postings included questions on all subjects. A non-medically trained moderator evaluated all postings and messages left on the site twice daily, and postings that included product labels were removed according to Finnish law on pharmaceutical product promotion. However, the sender was given the opportunity to re-send the posting if the product label was changed to a generic name. All questions were answered by the pediatrician (the author), even if they were outside the focus of the service. The pediatrician was employed on an hourly basis to the site, and at the time was also a research coordinator at a clinical postgraduate school at the University of Helsinki, Finland. The pediatrician answered questions once daily on weekdays. In addition, the moderator contacted the pediatrician regarding possible emergency-related questions potentially requiring an urgent response, in which case the parent received a notification to seek immediate treatment.

### Analysis

Typically, each posting included several questions. Postings were first analyzed for the number of questions, and then the questions were categorized according to the symptoms presented (allergies, nutrition, neurology, etc). Most of the questions did not include sufficient information to set a specific diagnosis. Therefore, categorization was performed in relation to the clinical entity the question belonged to. Infections were further categorized into subgroups. Otitis media (a middle ear infection) was the largest subgroup and as such was included in the analysis. Each of the other infection subgroups included only 1-30 questions, and thus these subgroups were omitted from further analyses, although included in the infection category.

Answers were evaluated by the author by reviewing the questions in each posting separately. If the answer did not include a referral to a health care service provider, the question was considered comprehensively answered. If the answer included a referral at a later time should certain criteria be met (such as in the case of new symptoms), the question was also considered comprehensively answered.

Analyses were performed using GraphPad Prism 6.0. Question data are presented as mean (SD). Proportions were compared using the chi-square test. User statistics were gathered from information in the Facebook user database, Page insights.

## Results

### Respondents

During the study, 245,533 independent Facebook users visited the site, an average of 727.0 (SD 2280.6) per day. During closure of the service (April to August 2012), there were an average of 17.4 (SD 48.8) daily visitors. In the average month, 75.0% of the visitors were women (SD 13.7). The largest visitor age group was 25-34 years in both women and men: 39.6% (SD 2.9) and 11.5% (SD 2.4) of all monthly visitors, respectively. Most of the visitors were inhabitants of the five largest cities in Finland (data not shown). By December 31, 2012, there were 12,328 Facebook likes since the beginning of the service.

### Postings

There were an average of 42.6 (SD 27.6) monthly postings from parents, 478 in total. Men sent a total of 9 postings. Each posting contained a mean 1.6 (SD 0.6) questions (N=768), 69.8 (SD 31.7) questions per month. Of all postings, 63.4% (303/478) were comprehensively answered.

### Questions

**Figure 2:**
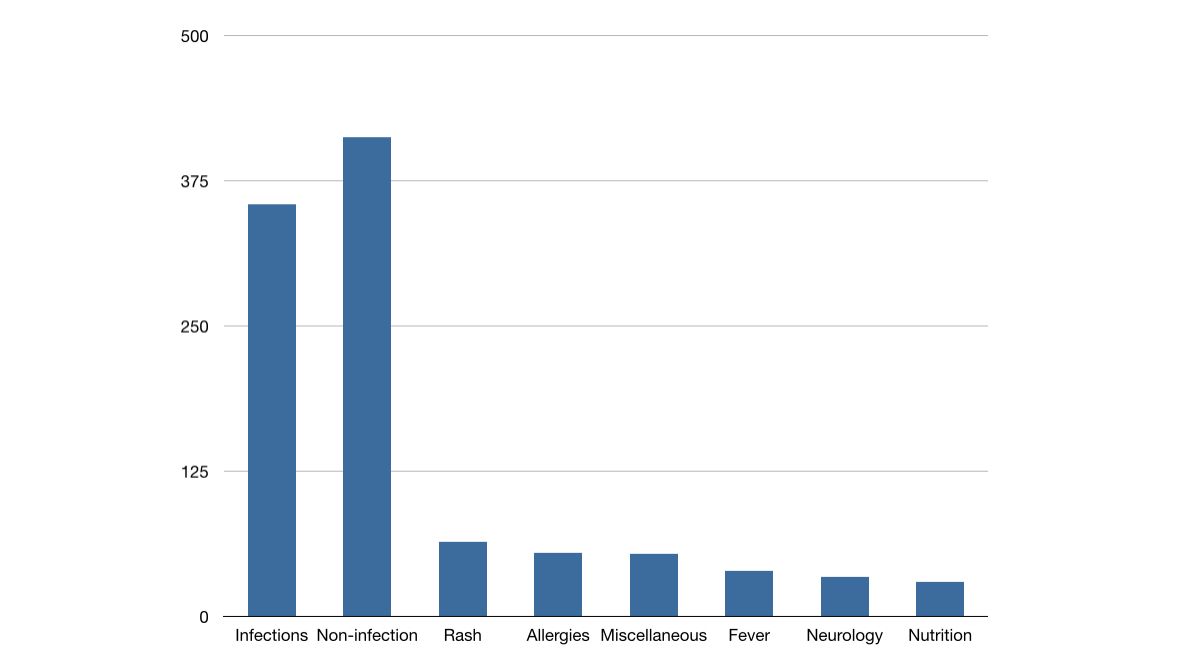
Number of questions categorized into topics (topics with less than 30 questions not shown).

Infections were the most common theme in questions (355/768, 46.2%), with otitis media–related questions being the most common subgroup of infections (69/768, 9.0% of all questions). There were 413 non–infection-related questions during the study period. The other symptom groups with more than 30 questions in the study period were non-infectious rash (64/768, 8.3%), allergies (55/768, 7.2%), fever (eg, help in choosing the right thermometer, 39/768, 5.1%), neurological symptoms (34/768, 4.4%), and nutrition (30/768, 3.9%, Figure 2). Questions that could not be categorized into the above entities were grouped into miscellaneous questions (54/768, 7.0%).

**Figure 3:**
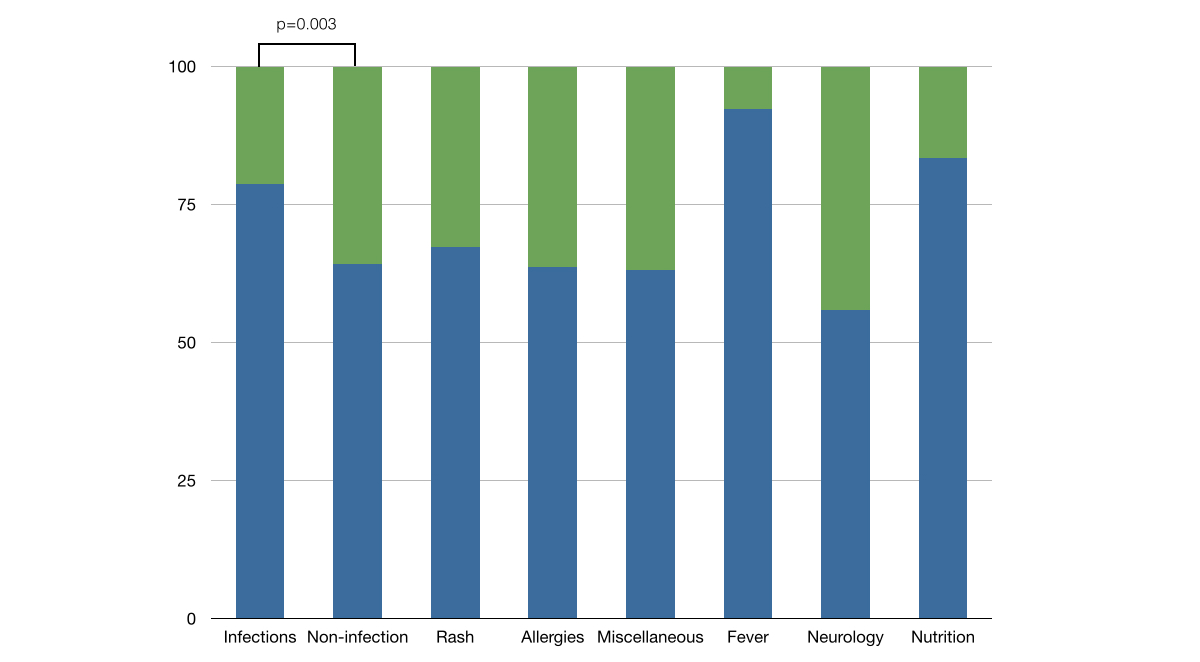
The proportion (%) of questions comprehensively answered (in blue) and the proportion requiring further contact with a health care worker (in green); P=.003 for the difference between questions comprehensively answered in the infections category and the non-infection category.

In general, only one exchange of postings occurred for each question. However, the need for referral was evaluated from each posting by the pediatrician. Questions relating to fever were most likely to be comprehensively answered (36/39, 92.3%) while questions relating to neurological symptoms were least likely (19/34, 55.9%; Figure 3). Questions were more likely to be comprehensively answered if they were related to infections (279/355, 78.6%) than questions related to non-infectious symptoms (265/413, 64.2%, chi-square=15.29, *P=*.003). Within the otitis media category, 85.5% of questions (59/69) were considered comprehensively answered.

**Figure 4:**
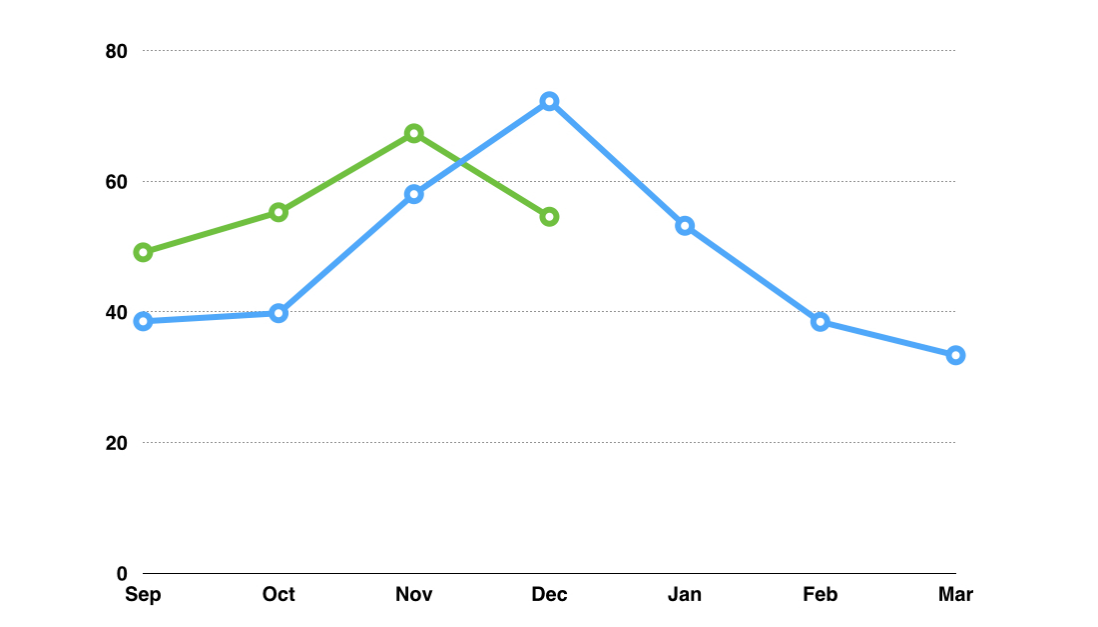
The percentage of questions relating to infections in 2011-2012 (blue) and 2012 (green).

Large seasonal variation in question topics was present. Infection-related questions dominated in the fall (Figure 4), tracing a similar pattern to reasons for ambulatory pediatric doctor’s visits.

## Discussion

### Principal Findings

This study provides an overview of a social media site for distributing child health information, a profile of the questions asked by parents, the proportions of questions comprehensively answered, and the volume of visitors on the site. On this site, which was aimed at parents of small children, personalized answers were an effective way of delivering information. Based on the number of questions considered comprehensively answered, the service may have reduced the need for further contacts with a health care service provider in more than half of the cases.

The service was directed to questions on child health. According to the site visitor profile, mostly women aged 29-34 years, it is likely that the audience was reached. The number of individual visits was almost 250,000. There was a great variation in the amount of daily visitors, but no trend could be seen to explain this. The number of postings generated by this audience was 478, so it is likely that the answer given to one posting was read by multiple users and information spread was greater than just one person, which is typical for this kind of media [11]. Evaluating the efficiency of social media is difficult [12], and choosing the correct key performance indicator is crucial [13]. In this study, the number of individual daily visitors was used alongside the number of actual postings. From these data, it is obvious that a need exists for a social media-based child health distribution site in Finland. In this study, it was not possible to assess the effect of the service on in-person visits to the health care service provider. This should be the focus of further studies.

Infections were the most common topic for parental questions. These are also the most typical cause for ambulatory pediatric doctor’s visit. Pediatric infectious diseases is a subspecialty within pediatrics, and this finding supports the fact that a specialist of this field would be the optimal person to work on a site with such a focus. In addition, the seasonal variation in questions on infections followed the general trend of pediatric infections in Finland. This underlines the fact that the questions presented were not just sporadically written, but followed general trends.

Social media is an interactive and sometimes face-to-face information distribution channel, and it is therefore considered a more reliable media than passive Internet pages [14]. It is unlikely that users evaluate critically the motives behind the information given [14]. Because the reliability of health information distributed through social media tools may be contradictory to medical guidelines [5] and because commercial interests may play a large role in delivering health information on social media [15], it is clear that information in line with current medical guidelines should also be easily accessible through social media channels. In specific fields of medicine, a site such as the one presented here could be used to effectively deliver guideline-derived health information to patients, for example by publicly funded institutes. For parents of small children, the trustworthiness of health information has been shown to be dependent on the expertise of the person answering the question. If the information is delivered by someone that the parents consider an expert, it is accepted as credible [10]. It is possible that the commercially funded service depicted on the site received a wide audience, because the questions were answered by a pediatrician.

The proportion of questions comprehensively answered was over 70%. Without this social media contact, many of these questions would likely have been presented to a health care service provider, possibly by making an appointment to see a doctor. Although it was not possible to evaluate in the study, the service may well have reduced the pressure on “well-baby” clinic personnel. Questions about infections were the most likely to be comprehensively answered, while questions concerning neurological symptoms were least likely to be comprehensively answered, demonstrating the difficulty in assessing neurological symptoms in general. In health information technology, effectiveness of the service is rarely evaluated [16]. On the current site, answers were evaluated by the pediatrician. In future studies, a patient-based evaluation should be included in the service.

When planning a site for health information, the scope has to be defined. If the scope is comprehensive and aimed at, for example, a wide array of child health issues, the expertise also needs to be more comprehensive. This is of course more costly. On the other hand, a narrow scope can also be recommended. The experience of this site suggests that even a narrow focus on infection-related questions only would have been functional especially because these questions were more likely to be comprehensively answered than non–infection-related questions. Here, the focus of the questions changed over time and the number of infection-related questions increased towards winter. Therefore, the expertise needed to answer the questions may vary over time. Another issue to be decided on is the lapse between questions and answers. It is recommended here that the lapse be fairly short. On this site, the lapse was 24 hours on weekdays and more than 2 days at weekends. This may have resulted in a lower number of visits and postings. The method here would serve its purpose best if it were focused narrowly and manned in such a way that the delay in answers was shorter.

### Limitations

One of the crucial concerns when launching a site is the possible discrepancy between the demand and the number of personnel answering to the demand, that is, the possibility of an overflow of questions. According to the data from this site, the number of questions is likely to be rather limited in comparison with the number of visitors. Hence, one question and answer may be viewed a multitude of times and the information delivered efficiently. While active social media participants are usually a minority [16], it is possible that the low activity on this site could have resulted from the site also being an advertisement. However, on average, one daily visitor would visit the site twice during the day (data not shown), which more likely suggests an active interest on the question-and-answer forum rather than on the static advertisement on the site.

Another possible limitation to the study is the fact that having a pharmaceutical product advertisement on the site may have skewed the focus of the questions presented on the site. As the product was an antipyretic drug, it is likely the possible effect was an increase in the number of questions on infections.

### Conclusions

There is a constant demand for social media–based child health information services. A social media health information site is likely to be well accepted if the consultant is a specialist and, when delivering child health information, possibly a pediatrician. On this site aimed at parents of children in Finland, the greatest number of questions was presented between September and January, and most of these concerned infections. Over half of the questions were estimated to be comprehensively answered, and most likely did not require further contact with a health care service provider. These factors should be taken into account when planning such a service.

## References

[1] Eysenbach G (2008). Medicine 2.0: social networking, collaboration, participation, apomediation, and openness. J Med Internet Res.

[2] Chou WY, Hunt YM, Beckjord EB, Moser RP, Hesse BW (2009). Social media use in the United States: implications for health communication. J Med Internet Res.

[3] Facebook company website.

[4] (2010). Corporation TNR.

[5] Chou WY, Prestin A, Lyons C, Wen KY (2013). Web 2.0 for health promotion: reviewing the current evidence. Am J Public Health.

[6] Syed-Abdul S, Fernandez-Luque L, Jian WS, Li YC, Crain S, Hsu MH, Wang YC, Khandregzen D, Chuluunbaatar E, Nguyen PA, Liou DM (2013). Misleading health-related information promoted through video-based social media: anorexia on YouTube. J Med Internet Res.

[7] Thackeray R, Neiger BL, Smith AK, Van Wagenen SB (2012). Adoption and use of social media among public health departments. BMC Public Health.

[8] McGowan BS, Wasko M, Vartabedian BS, Miller RS, Freiherr DD, Abdolrasulnia M (2012). Understanding the factors that influence the adoption and meaningful use of social media by physicians to share medical information. J Med Internet Res.

[9] Hakulinen-Viitanen T, Pelkonen M, Haapakorva A (2005). Maternity and child health care in Finland. Reports of the Ministry of Social Affairs and Health.

[10] Stroever SJ, Mackert MS, McAlister AL, Hoelscher DM (2011). Using social media to communicate child health information to low-income parents. Prev Chronic Dis.

[11] Thackeray R, Crookston BT, West JH (2013). Correlates of health-related social media use among adults. J Med Internet Res.

[12] Hussain AA (2011). Meaningful use of information technology: a local perspective. Ann Intern Med.

[13] Neiger BL, Thackeray R, Van Wagenen SA, Hanson CL, West JH, Barnes MD, Fagen MC (2012). Use of social media in health promotion: purposes, key performance indicators, and evaluation metrics. Health Promot Pract.

[14] Metzger MJ, Flanagin AJ (2011). Using Web 2.0 technologies to enhance evidence-based medical information. J Health Commun.

[15] Gregerson J (2012). Truth, lies, and rumors in the media: consider the source. J Acad Nutr Diet.

[16] Moorhead SA, Hazlett DE, Harrison L, Carroll JK, Irwin A, Hoving C (2013). A new dimension of health care: systematic review of the uses, benefits, and limitations of social media for health communication. J Med Internet Res.

